# Foxp3^+^ Regulatory T Cells Are Required for Recovery from Severe Sepsis

**DOI:** 10.1371/journal.pone.0065109

**Published:** 2013-05-28

**Authors:** Franziska Kühlhorn, Matthias Rath, Katrin Schmoeckel, Katharina Cziupka, Huu Hung Nguyen, Petra Hildebrandt, Thomas Hünig, Tim Sparwasser, Jochen Huehn, Christian Pötschke, Barbara M. Bröker

**Affiliations:** 1 Institute of Immunology and Transfusion Medicine, University Medicine Greifswald, Greifswald, Germany; 2 Department of Surgery, University Medicine Greifswald, Greifswald, Germany; 3 Interfaculty Institute of Genetics and Functional Genomics, Department of Functional Genomics, University of Greifswald, Greifswald, Germany; 4 Institute of Virology and Immunobiology, University of Würzburg, Würzburg, Germany; 5 Institute of Infection Immunology, TWINCORE, Centre for Experimental and Clinical Infection Research, Hannover, Germany; 6 Helmholtz Centre for Infection Research, Braunschweig, Germany; University Hospital Jena, Germany

## Abstract

The role of regulatory T cells (Tregs) in bacterial sepsis remains controversial because antibody-mediated depletion experiments gave conflicting results. We employed DEREG mice (DEpletion of REGulatory T cells) and a caecal ligation and puncture model to elucidate the role of CD4^+^Foxp3^+^ Tregs in sepsis. In DEREG mice natural Tregs can be visualized easily and selectively depleted by diphtheria toxin because the animals express the diphtheria toxin receptor and enhanced green fluorescent protein as a fusion protein under the control of the *foxp3* locus. We confirmed rapid Treg-activation and an increased ratio of Tregs to Teffs in sepsis. Nevertheless, 24 h after sepsis induction, Treg-depleted and control mice showed equally strong inflammation, immune cell immigration into the peritoneum and bacterial dissemination. During the first 36 h of disease survival was not influenced by Treg-depletion. Later, however, only Treg-competent animals recovered from the insult. We conclude that the suppressive capacity of Tregs is not sufficient to control overwhelming inflammation and early mortality, but is a prerequisite for the recovery from severe sepsis.

## Introduction

Sepsis remains a major cause of death in intensive care units worldwide [1]. Especially postoperatively acquired abdominal sepsis due to intestinal leakage is still associated with a very high lethality of about 60% [2].

During the last years, increasing attention has been directed at the role of the adaptive immune system, since it became apparent that T cells can strongly influence the course of the disease even in the first days of sepsis [3]–[6]. One T cell subpopulation became a major focus of interest: natural regulatory T cells (Tregs). These cells have been shown to be of central importance for the maintenance of immune homeostasis and self-tolerance. Their ablation leads to catastrophic autoimmune disease in mice and humans [7]–[10]. During infection Tregs can prevent excessive immunopathology and increase survival under some conditions [11]–[13], whereas in other circumstances the dampening effects of natural Tregs may interfere with protective immune responses [14]–[17]. Thus Tregs are a double-edged sword in infection, limiting inflammation and collateral tissue damage at the price of interference with bacterial clearance [15], [17]. Therefore, as a prerequisite for possible therapeutic intervention, it is important to understand whether Tregs have a beneficial or deleterious impact on the outcome of abdominal sepsis.

Yet, studies on Treg function in sepsis using CD25 to characterize Tregs yield conflicting results. In the caecal ligation and puncture (CLP) model of murine sepsis, Heuer et al. reported improved survival after adoptive transfer of small numbers of *ex vivo* activated CD4^+^CD25^+^ Tregs [18]. Following Treg depletion with anti-CD25 mAbs, other groups observed no effect [19], [20] or even improved survival in murine sepsis [21]. One has to bear in mind that CD25 is not exclusively expressed on Tregs but is rapidly induced on naïve T cells and T effector cells (Teffs) upon activation. On the other hand, a significant proportion of Foxp3^+^ Tregs does not express CD25 [22]–[24]. The anti-CD25 antibody (PC61), which has been used in many studies on Tregs, will not deplete this Foxp3^+^CD25^−^ subpopulation and is only partially efficient in depleting CD25^+^ Tregs [25]. Furthermore, the antibodies remain in the system for several days and could then affect Teffs, which become activated within hours upon sepsis induction [3], [26].

To overcome these experimental constraints, researchers have recommended using the transcription factor Foxp3 as a marker for Tregs when exploring how these cells shape the immune response in sepsis [17], [27]. It has been well documented, that Foxp3 is selectively expressed by CD4^+^ regulatory T cells in the murine system [28]–[33] as well as by a small subpopulation of CD8^+^ cells. The latter have been attributed with a role in autoimmune disorders [31]–[35], graft-versus-host disease [36]–[38] and they suppressed immune responses against malignancies [39], [40].

Foxp3^+^ cells are defined as *bona fide* Tregs in this manuscript. We have taken advantage of DEREG mice (DEpletion of REGulatory T cells), which express a primate diphtheria toxin receptor fused to enhanced green fluorescent protein (eGFP) under the control of the *foxp3* promoter [9]. This enabled us to visualize Foxp3^+^ Tregs *ex vivo* and to selectively deplete them *in vivo*. We employed the caecal ligation and puncture (CLP) model of sepsis to examine changes in Treg phenotype and function in generalized bacterial infection. Similar to the human disease, CLP causes rapid systemic inflammation as a consequence of the continuous dissemination of endogenous gut bacteria [41].

After sepsis induction, Tregs were rapidly activated systemically and showed enhanced suppressive capacity *in vitro*. However, depletion of Foxp3^+^ Tregs did not change early mortality, but decreased survival in late disease. Adoptive transfer of pre-activated Tregs before sepsis induction did not improve the outcome.

## Materials and Methods

### Animal experiments and ethics statement

DEREG mice were on a C57BL/6 genetic background. These mice are heterozygous for a diphtheria toxin receptor-eGFP construct under the control of the *foxp3* promoter [9]. C57BL/6 wild type (WT) mice served as controls. The mice were housed in a conventional, temperature-controlled animal facility with a 12-hour light/12-hour dark cycle and provided with food and water *ad libitum*. All experiments were performed according to the German animal safety regulations and approved by the animal ethics committee of the local animal protection authority (LALLF, Landesamt für Landwirtschaft, Lebensmittelsicherheit und Fischerei Mecklenburg-Vorpommern). During experimental procedures, the animals were provided with food and water ad libitum. All efforts were made to minimize suffering.

### Peritoneal sepsis model – caecal ligation and puncture (CLP)

Female mice, 8–12 weeks old, were anaesthetized with Ketamin/Xylazin (100 mg/10 mg per kg bodyweight). The caecum was ligated 1.3 cm above the distal ending and punctured once at the anti-mesenteric side with an 18 G needle. The mice received 0.3 ml 0.9% NaCl i.p. for volume substitution.

Survival and disease severity were monitored every 3 h for 72 h by an observer who was blinded for the treatments applied. Disease severity was scored on the basis of (1) general appearance, (2) breathing frequency, (3) spontaneous and (4) provoked behaviour. Scoring points from 0 =  healthy to 3 =  severe alteration were given for each item and then summed up [42]. If the mice reached a severity score that indicated a disease point of no return, these mice were euthanized by cervical dislocation under deep anaesthesia.

### Depletion of regulatory T cells

For *in vivo* Treg depletion, 1 µg diphtheria toxin (DT; Merck, Darmstadt, Germany) dissolved in 100 µl phosphate buffered saline (PBS) was administered intravenously to DEREG mice on days −2 and −1 before the CLP operation. Depletion of the Treg cell population was confirmed by flow cytometry and histology and reached an efficiency of about 95% in the spleen, mesenteric lymph node, thymus, and blood (Figure S1).

### Determination of the bacterial load

24 h after CLP, the mice were put into deep anaesthesia and sacrificed by cervical dislocation. Spleen and liver were obtained. The homogenized organ suspensions were incubated on Columbia blood agar (Becton Dickinson, Heidelberg, Germany) for 22 h at 37°C. Bacterial colonies were enumerated and related to organ weight.

### Antibodies for flow cytometry

Peritoneal lavage and spleen cell suspensions were obtained as described before [3]. The following fluorochrome-labelled or unlabelled antibodies were purchased from BD Biosciences (Heidelberg, Germany): αCD4 (RM4–5), αCD69 (H1.2F3), hamster αCTLA-4 (UC10-4F10-11), αLy6G (1A8), αCD11b (M1/70), a biotinylated cocktail of mAbs against Armenian and Syrian hamster IgG, and αCD11c (HL3). Antibodies directed against MHC-II (M5/114.15.2), CD3 (145-2C11), F4/80 (BM8), CD25 (PC61.5) and CD19 were from eBioscience (Hatfield, UK). For intracellular Foxp3 staining, we used Foxp3-mAb (3G3) and the FoxP3-Staining Buffer Set from MiltenyiBiotec (Bergisch Gladbach, Germany). Streptavidin-Alexa647 was obtained from Invitrogen.

### Cytokine expression

To determine serum concentrations of IL-6, TNFα, MCP-1, IFN-γ, IL12p70, and IL-10, a mouse inflammation cytometric bead array kit (BD, San Diego, USA) was used according to the manufacturer's instructions.

### Isolation of Tregs, Teffs and antigen presenting cells

Splenic CD4^+^ T cells from DEREG mice were negatively selected by magnetic cell sorting (MACS) with the CD4^+^ T cell isolation Kit II, MACS MiltenyiBiotec. Afterwards the eGFP-expressing Tregs and the eGFP-negative Teffs were sorted with a fluorescence-activated cell sorter (FACS), FACSAria. In general we obtained Tregs of about 98% purity and >70% viability. Teff purity was about 95% and viability was 94% (Figure S2 A and B). CD11c^+^ antigen-presenting cells (APC) with a purity of 93% (Figure S2C) were isolated with the CD11c-Microbeads-Kit, MiltenyiBiotec. Before seeding the APC onto culture plates, they were irradiated with 30 Gray.

### Adoptive transfer of activated Tregs

DEREG mice received 250 µg of a superagonistic anti-CD28 monoclonal antibody (αCD28 SA, clone D665) in the lateral tail vein three days before they were sacrificed under deep anaesthesia by cervical dislocation and their spleens explanted. αCD28 SA has been shown to increase Treg numbers and activation status [43]. *In vivo* pre-activated Tregs as well as Tregs and Teffs from untreated animals were isolated as described above. 3×10^5^ cells were transferred into the lateral tail vein of female C57BL/6 mice directly before CLP.

### Proliferation assay

5×10^4^ isolated Teffs were co-incubated for 72 h with 1×10^4^ irradiated APC, varying numbers of Tregs and 1 µg/ml soluble anti-CD3 antibody (eBioscience). ^3^H-Thymidine was added for the last 17 h of culture, and incorporation was measured in counts per minute (cpm).

### Statistical analysis

Statistical analyses were performed using GraphPad Prism 5 for Windows (GraphPad software, San Diego, CA, USA). Survival was analysed with Kaplan-Meier survival curves and compared with the log rank test. Bacterial loads, cytokine concentrations and expression of activation markers were assessed for significant differences using ANOVA with Bonferroni posttest for selected pairs. Differences in the suppressive capacity of Tregs isolated from septic vs non-septic animals were compared with a non-parametric t-test. P-values <0.05 were considered to be significant.

## Results

### Activation of Foxp3^+^ Tregs and Foxp3^−^ Teffs in sepsis

**Figure 1:**
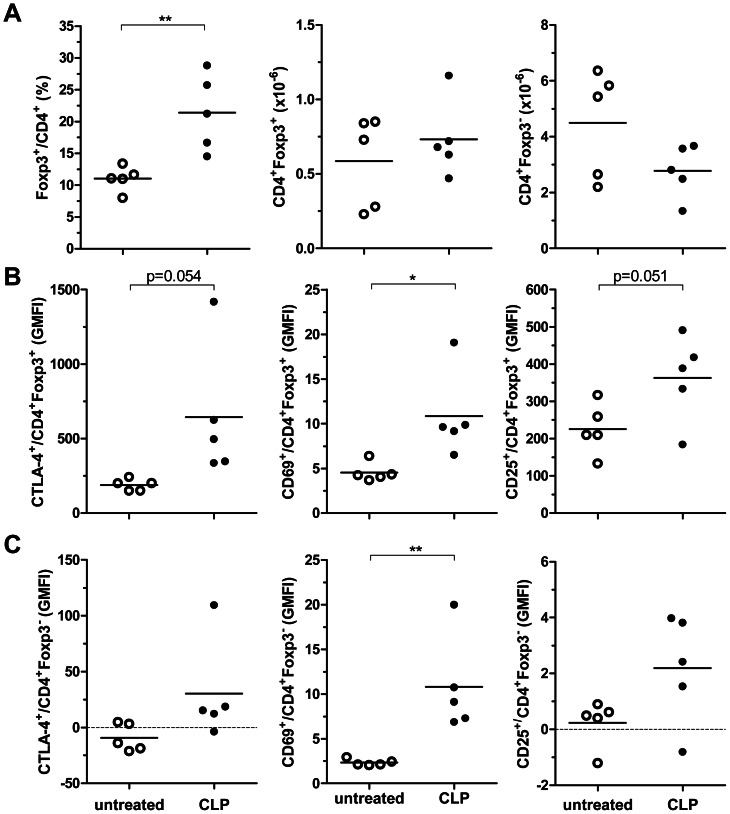
CLP rapidly activated CD4^+^T cells and increased the percentage of the Foxp3^+^ Treg subpopulation. DEREG mice were subjected to 18G CLP or left untreated. Twenty-four hours later the percentage of Foxp3^+^ cells in the CD4^+^ T cell subpopulation and the absolute numbers of CD4^+^Foxp3^+^ and CD4^+^Foxp3^−^ cells of the spleen were determined (A). In addition, the expression of the activation markers CTLA4, CD69, and CD25 on Foxp3^+^ Tregs (B) and Foxp3^−^ Teffs (C) was assessed (GMFI, geometric mean fluorescence intensity). The means are indicated. n = 5 mice/group. * p<0.05; ** p<0.01.

To address the impact of sepsis on natural Tregs, we subjected DEREG mice to CLP and measured the proportion of Foxp3^+^ cells in the CD4^+^ T cell population. Twenty-four hours after sepsis induction, the percentage of CD4^+^Foxp3^+^ splenocytes was significantly increased (Figure 1A). As previously observed in human and murine sepsis [44], [45], this shift in the Treg/Teff ratio was probably due to the relative resistance of Tregs to apoptosis [44], [46] rather than to Treg expansion because the total number of CD4^+^Foxp3^+^ Tregs in the spleen was not significantly altered (data not shown). In addition, Tregs strongly upregulated the markers CTLA-4, CD69 and CD25, indicating that they were activated within hours after sepsis induction (Figure 1B). The Foxp3^−^ Teffs were also rapidly activated (Figure 1C).

### Tregs increased their suppressive potential in sepsis

**Figure 2:**
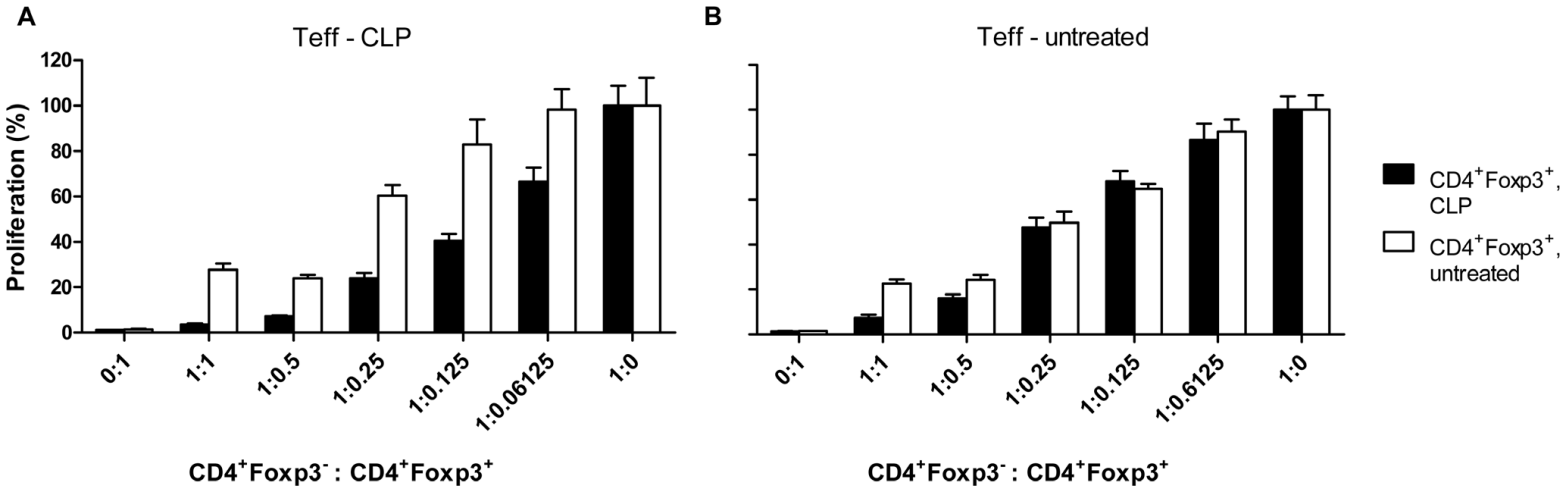
Foxp3^+^ Tregs maintained their suppressive capacity after CLP. CD4^+^Foxp3^−^ Teffs (5×10^4^) were isolated 24 h after 18G CLP (A) or from untreated C57BL/6 mice (B) and co-incubated at the given ratios with CD4^+^Foxp3^+^ Treg isolated from CLP-treated (filled bars) or untreated DEREG mice (open bars). They were cultured for 72 h in the presence of 1×10^4^ irradiated APCs and 1 µg/ml soluble anti-CD3 antibody. ^3^H-Thymidine was added for the last 17 h of culture, and incorporation was measured as counts per minute (cpm). Cpm of Teff without Treg was set to 100%. Means +/− SEM are indicated. One of two independent experiments with similar results is shown. Differences between the suppressor activity of Tregs from septic and non-septic mice were tested for significance at each effector-suppressor ratio (t-test). * p<0,5; *** p<0.001.

Some groups have reported enhanced suppressor activity of Tregs 24 h after sepsis induction [19], while other groups did not find changes in suppressive Treg activity earlier than three days after sepsis [20], [47]. We observed significantly enhanced suppressor activity in *in vitro* co-culture assays already 24 h after CLP. Foxp3^+^ Tregs from septic mice potently suppressed the activation of Teffs from both untreated and septic animals. The relative increase of suppressor activity was most pronounced when Teffs from septic mice were targeted (Figure 2) clearly showing that during sepsis Teffs did not become inherently resistant to the inhibitory effects of Tregs.

### Treg depletion reduced sepsis survival

To test how these activated suppressive Foxp3^+^ Tregs influence the outcome of sepsis, we depleted Tregs from the system before sepsis induction. In contrast to other studies worldwide, we did not use a depleting antibody, but treated DEREG mice with DT to selectively deplete Tregs. DT-treated C57BL/6 WT mice served as controls.

**Figure 3:**
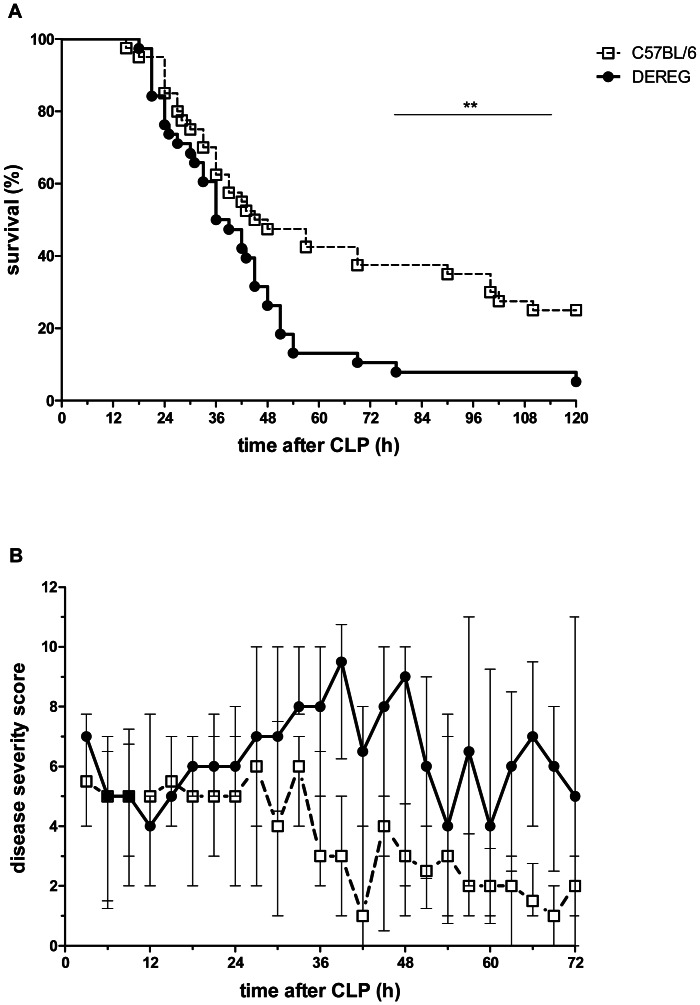
Treg-depletion augmented disease severity and decreased survival in CLP-treated mice. DEREG and C57BL/6 mice pre-treated with DT (1 µg in 100 µl PBS i.v. on days −2 and −1) were subjected to CLP. Survival was monitored for 5 days (A) and disease severity was scored for 3 days (B) by an observer blinded for the group assignment. Data from four independent experiments with similar results are summarized (each experiment involved 8–11 mice/group). Median and interquartile range of the disease severity score are shown in panel B; ** p<0.01.

Both groups showed similar survival kinetics until 36 h post-CLP (Figure 3A). Thereafter, the curves split. 25% of Treg-competent but only 5% of Treg-depleted animals survived. This was mirrored by the disease severity. After 36 h Treg-competent animals began to recover, while Treg-depleted animals continued to deteriorate (Figure 3B).

### Treg depletion did not alter peritoneal leukocyte migration, bacterial clearance or serum cytokine production

**Figure 4:**
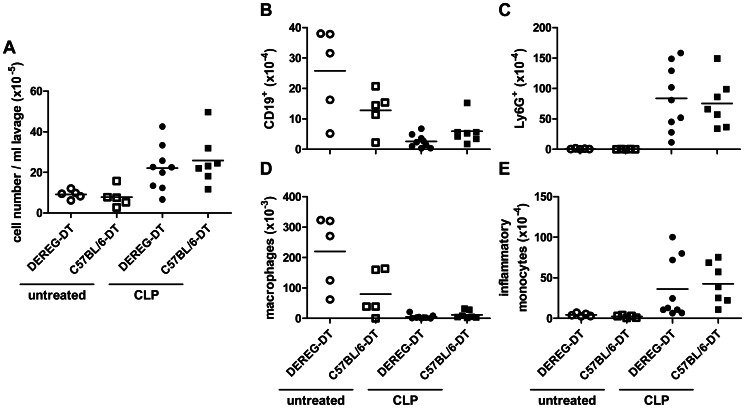
Depletion of Tregs did not alter cell migration into the peritoneum. DEREG and C57BL/6 mice pre-treated with DT (1 µg in 100 µl PBS i.v. on days −2 and −1) were subjected to CLP (n = 7–9 mice/group) or left untreated (n = 5 mice/group). Cells from the peritoneal cavity were harvested by peritoneal lavage 24 h after CLP. The total number of cells was enumerated and related to volume of recovered lavage fluid (A). The numbers of B cells (CD19^+^) (B), neutrophils (Ly6G^+^) (C), peritoneal macrophages (F4/80^+^CD11b^+^MHCII^+^) (D) and inflammatory monocytes (F4/80^-^CD11b^+^MHCII^lo^) (E) was assessed via flow cytometry. Means are shown.

**Figure 5:**
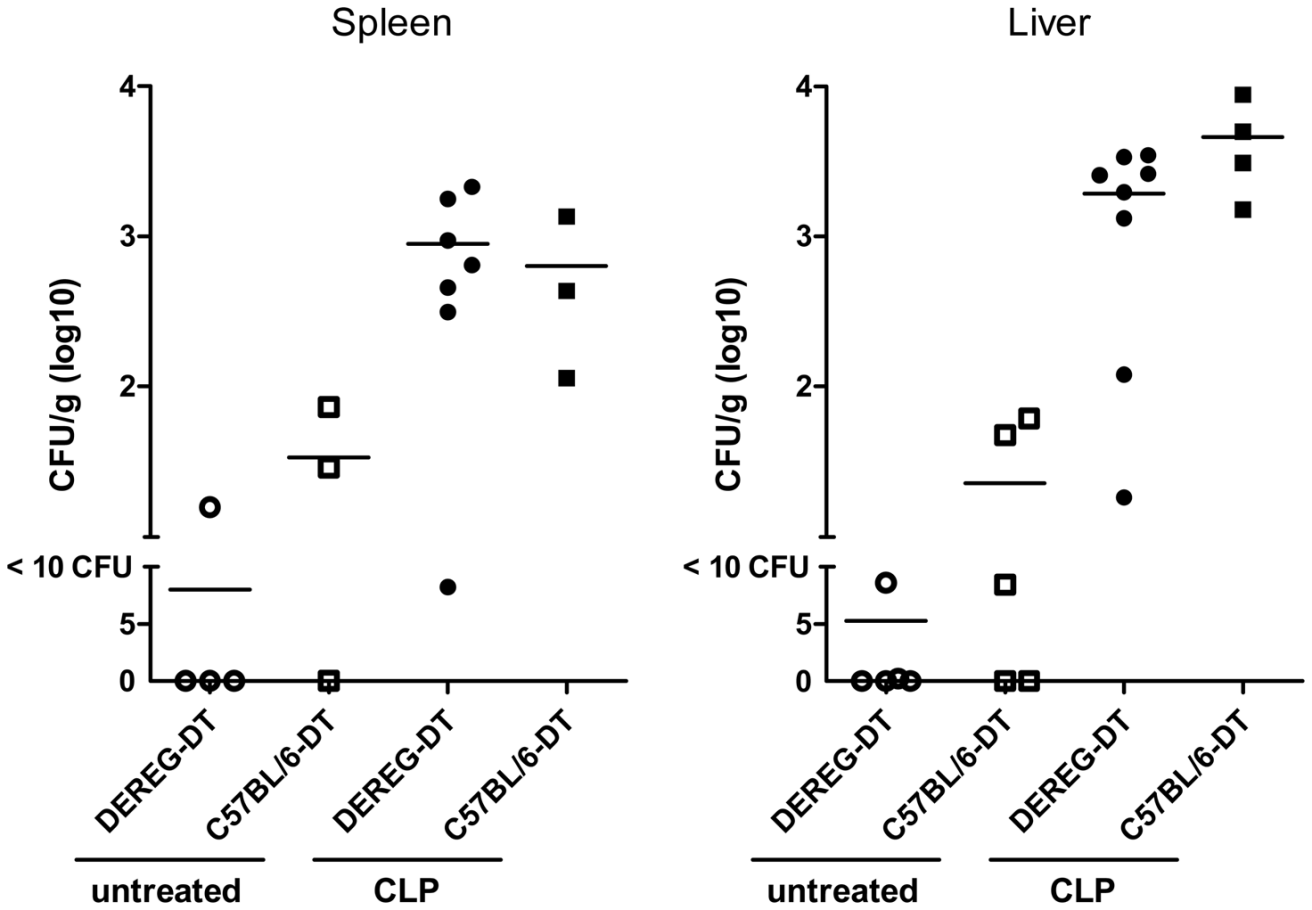
No influence of Treg depletion on bacterial dissemination in CLP-treated mice. DEREG and C57BL/6 mice pre-treated with DT (1 µg in 100 µl PBS i.v. on days −2 and −1) were subjected to CLP (n = 3–7 mice/group) or left untreated (n = 3–5 mice/group). Liver and spleen were recovered from Treg-depleted and non-depleted mice 24 h after CLP. Tissue homogenates were incubated on Columbia blood agar for 22 h at 37°C. Colony-forming units (CFUs) were related to organ weight. Means are shown.

**Figure 6:**
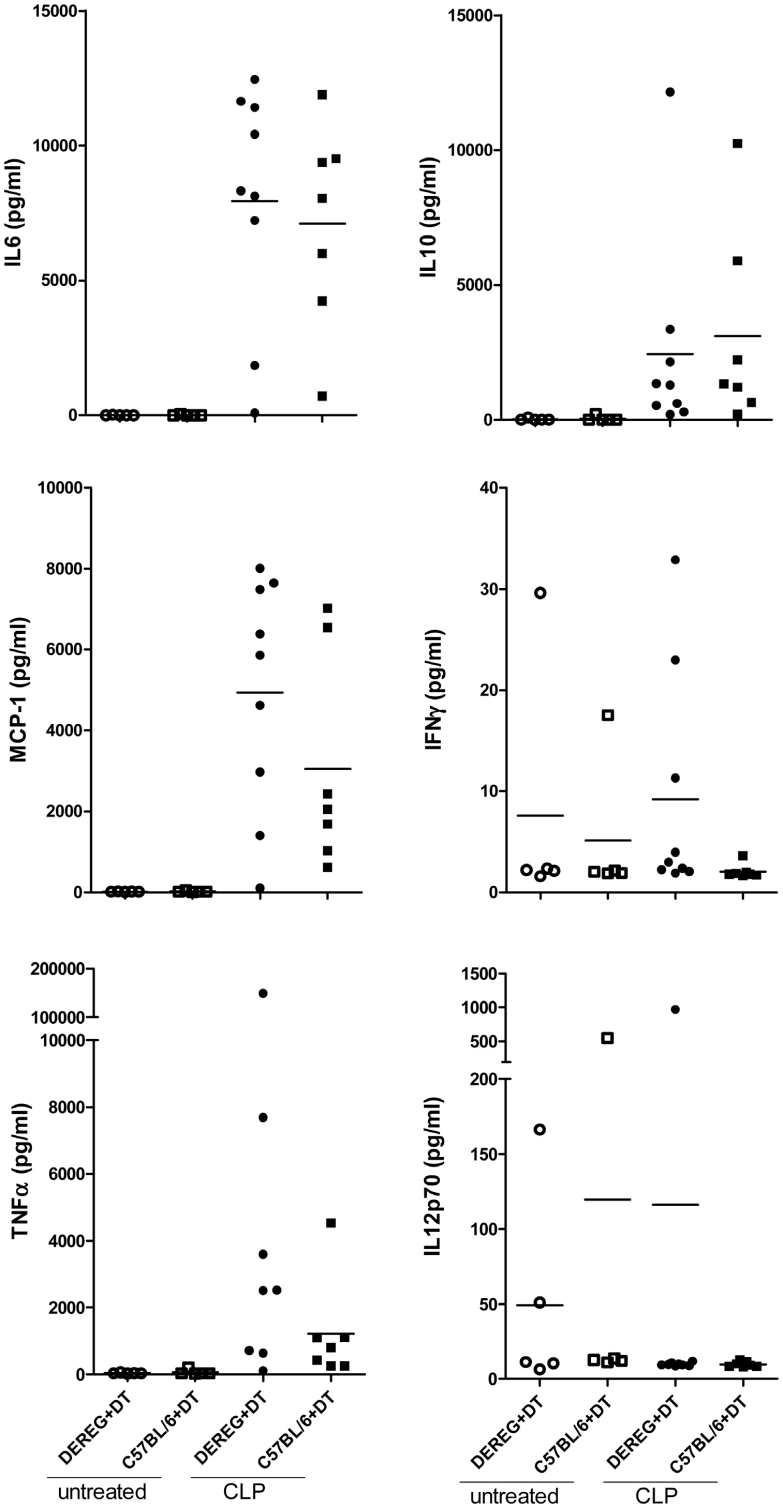
Serum cytokines in CLP-treated mice. DEREG and C57BL/6 mice pre-treated with DT (1 µg in 100 µl PBS i.v. on days −2 and −1) were subjected to CLP or left untreated. Twenty-four hours later serum cytokine concentrations (IL6, IL10, MCP-1, IFNγ, TNFα and IL12p70) increased in septic animals, but there were no differences between Treg-depleted and non-depleted septic mice. Means are indicated; n = 5–9 mice/group.

Twenty-four hours after CLP we measured an increase of peritoneal cell content (Figure 4A). This was mainly due to an increase of absolute neutrophil and inflammatory monocyte counts, while flow cytometric analysis revealed a decrease of B cells and resident peritoneal macrophages. There was no difference between Treg-depleted and non-depleted mice (Figure 4B–E). The bacterial loads in spleen and liver (Figure 5) as well as the proinflammatory serum cytokine concentrations, which strongly increased following CLP, were also indistinguishable between Treg-depleted and non-depleted mice (Figure 6).

### Adoptive transfer of pre-activated Tregs did not alter survival after sepsis

**Figure 7:**
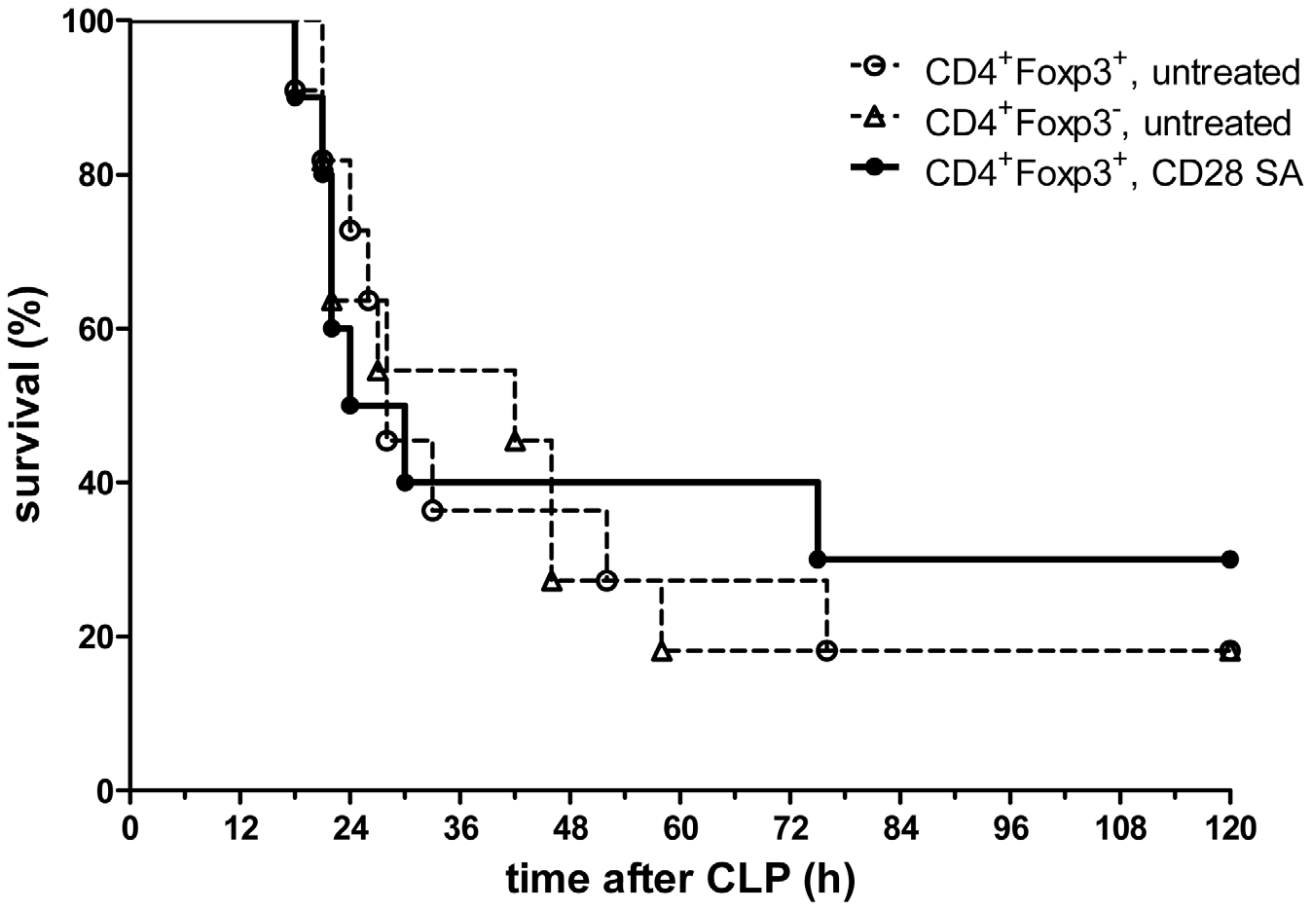
Adoptive transfer of in vivo activated Tregs did not improve survival after CLP. DEREG mice received 250 µg CD28 SA i.v. three days before their spleens were explanted and *in vivo* pre-activated CD4^+^Foxp3^+^ Tregs were isolated. 3×10^5^ of those cells were adoptively transferred into female C57BL/6 recipients (i.v.). (i) Untreated CD4^+^Foxp3^+^ and (ii) untreated CD4^+^Foxp3^−^ cells were transferred as controls. 18G CLP was performed immediately after the cell transfer and survival was monitored every three hours for three days. The Kaplan-Meier survival curves are shown. n = 10–11 mice/group.

In sepsis Tregs became activated and showed enhanced suppressive capacity. However, *in vivo* they were not able to improve mortality at early time points. We wondered whether an adoptive transfer of pre-activated Tregs could support endogenous Tregs in suppressing hyperinflammtion in the early phase. Since activation of purified Tregs *in vitro* led to pronounced apoptosis ([48] and not shown), we pre-activated Tregs *in vivo* by administering 250 µg of a CD28 superagonistic antibody to DEREG mice. Within three days, this treatment increased the number of splenic Foxp3^+^ Tregs threefold and strongly activated the cells (Figure S3A, B). In a two-step isolation procedure (see materials and methods), the eGFP^+^ splenic Tregs were enriched to >95% purity. They were >70% viable and had increased suppressive potential when co-cultured with Foxp3^−^ Teffs from untreated animals (Figure S3C). *In vivo* pre-activated Tregs (3×10^5^) were adoptively transferred (i.v.) into WT mice immediately after purification and directly before CLP-surgery. Control mice received untreated Tregs or Teffs. The adoptively transferred pre-activated Tregs did not significantly alter CLP survival (Figure 7).

## Discussion and Conclusion

Experiments with CD25-specific antibodies for depleting Foxp3^+^ Tregs have yielded conflicting results in sepsis studies [19]–[21]. Conclusions from these studies are limited because, on one hand activated Teffs also express CD25, and on the other hand many Tregs do not express CD25. Therefore, we opted for DEREG-mice who at present are the best available experimental system to visualize Foxp3^+^ Tregs and deplete them selectively without the need for antibodies.

In DEREG animals, depletion of Foxp3^+^ Tregs by DT was almost complete (92–98%). Not surprisingly it has been shown that Foxp3^−^ Tregs are unaffected [9], [49], [50], which limits the scope of this study. Control WT mice were not affected by DT treatment. Using a similar sepsis model, the colon ascendens stent peritonitis [42], we have compared two types of controls, WT mice receiving DT and DEREG mice treated with saline only, and did not observe differences regarding immune cell immigration into the peritoneum, bacterial dissemination nor serum cytokine concentrations (unpublished observation).

The vast majority of the depleted Foxp3^+^ cells are CD4^+^ *bona fide* Tregs. A contribution of Foxp3^+^ CD8^+^ T cells to the resolution of sepsis symptoms cannot be excluded but is probably minor, as they make up 0.1–0.4% of the CD8^+^ T cell pool and only 2% of the total Foxp3^+^ population [51]. Furthermore, Foxp3^+^ CD8^+^ T cells apparently lack potent suppressive capacity *in vitro*, even though they share phenotypic features with classical CD4^+^ Tregs [51].

An additional concern was the potential influence of cell death. Although the clinical picture of diphtheria is associated with necrosis, DT acts by inhibiting protein biosynthesis and, as a result, apoptotic cell death without inflammation [26], [52]. Hotchkiss et al. showed that apoptotic cells have a mild negative influence on sepsis outcome, which was accompanied by a reduction of IFNγ secretion following *ex vivo* stimulation of splenocytes and also by severely impaired bacterial eradication [53]. None of this was observed in our system following Treg depletion. Bacterial dissemination was not affected during the first 24 h of sepsis, and serum concentrations of inflammatory cytokines, including IFNγ, either did not change, or they were in tendency increased in Treg-depleted septic animals.

In our CLP model of severe abdominal sepsis (75% lethality at day 5), Foxp3^+^ Tregs became activated within 24 hours and displayed enhanced *in vitro*-suppressive function, which corroborated our previous findings and those of Scumpia et al. [3], [19]. Moreover, Teffs from septic animals did not become inherently resistant to the inhibitory effects of Tregs as they were even more readily suppressed *ex vivo* than Teffs from untreated controls. In agreement with the observations of other groups, the ratio between Foxp3^+^ Tregs and Foxp3^−^ Teff cells was significantly shifted towards Tregs [19], [27], [44], [54], but the absolute numbers of splenic Tregs did not change. Apparently, the increase in the Treg proportion in sepsis is mainly due to their relative resistance to lymphocyte apoptosis as has been well documented in humans and mice [44], [45], [55]–[58]. In contrast, many Teffs underwent cell death. It may be speculated that the increased responsiveness to Treg inhibition *ex vivo* characterizing Teffs from septic animals could be the result of Teff selection by cell death. Peripheral conversion of Teffs into induced Tregs may also increase the relative Treg numbers. Under our conditions, however, this appears less likely because *in vitro* experiments have shown that Treg induction takes more than 48 h [59]–[61]. Also, Foxp3 expression in induced CD8^+^ Tregs is reported to be rather unstable [51].

Thus one day after sepsis induction Foxp3^+^ Tregs were strongly activated, increased in proportion relative to Teffs and their *in vitro*-suppressive capacity was enhanced. Nevertheless, Treg depletion did not alter the early course of the disease in our severe CLP model. Later, however, Foxp3^+^ Tregs were protective and improved survival from only 5% in depleted animals to 25% in Treg-competent mice. Between one and two days after CLP, Treg-competent survivors began to recover, whereas disease symptoms continued in the Treg-depleted animals.

Why did Tregs not alter survival on the first day after CLP or dampen the proinflammatory cytokine response in this study, although, after removal from the septic microenvironment and three days in cell culture, they displayed enhanced suppressor activity? In sepsis the organism is flooded with large amounts of bacterial compounds that function as microorganism-associated molecular patterns (MAMPs). Such ligands of pattern recognition receptors on immune cells may modulate immune suppression either directly or indirectly. Regarding direct effects, there is controversy about how TLR ligation affects Treg function [62]–[65]. This can be (partially) explained by the kinetics of events. For example, TLR2 triggering transiently reduced the suppressive capacity of Tregs, but after the signal had declined, the cells regained suppressor activity [66], [67]. Even more important for Treg function could be indirect effects of inflammatory cytokines, such as TNFα and IL6, which are released by immune cells following MAMP stimulation [68]. In combination these cytokines impair the suppressive function of Tregs [69]–[71]. Upon removal of the microbial compounds and cytokines, the suppressor activity of Tregs is restored or even enhanced [69]. On the other hand, activated Tregs might face overwhelming inflammation in sepsis rendering immune cells refractory to suppression, or the activated Tregs are simply outnumbered by activated (innate) immune cells.

We therefore adoptively transferred activated Tregs similar to Heuer et al. [18], who used *in vitro*-activated Tregs. Since in our hands isolated Foxp3^+^ Tregs rapidly died in cell culture corroborating the reports of Zeng and co-workers [48], we decided to activate the Tregs *in vivo* using a CD28 superagonistic monoclonal antibody [43]. In this manner we avoided the well-documented effects of necrotic and/or apoptotic cells on the course of the disease [53], [72], [73]. After pre-activation in DEREG mice, the brightly fluorescent Foxp3^+^ cells could be enriched to very high purity and immediately transferred into the experimental animals at good viability. The transferred cells had a remarkable suppressive potential in cell culture (Figure S3), but they did not alter the fate of animals subjected to CLP. Whereas Heuer and co-workers reported a protective effect with as few as 3×10^4^ *in vitro* activated CD4^+^CD25^+^ Tregs [18], in our experiments even the transfer of 3×10^5^ *in vivo* pre-activated Foxp3^+^ Tregs did not improve survival. This could be due to a loss of the pre-activated state of exogenous Tregs upon adoptive transfer into septic animals or a refractory state of endogenous immune cells.

It appears that in severe sepsis, activated Foxp3^+^ Tregs were initially overwhelmed by inflammatory stimuli. Later in the disease, however, Tregs had a significant protective effect. These findings support the model of “tuned suppression”, namely the idea that in a highly inflammatory microenvironment even activated Tregs transiently lose their suppressive influence, thus enabling a powerful antimicrobial defence [69], [74]. Later, when the initial cytokine storm has significantly decreased, Tregs regain their suppressive influence and dampen inflammatory effector mechanisms thereby minimizing host tissue damage [75]–[79]. Our results show that this is important for the recovery from severe sepsis.

In summary, Treg-depletion worsened late survival, hence implying a beneficial role for Foxp3^+^ Tregs in severe sepsis.

## Supporting Information

Figure S1 **Efficiency of in vivo Treg depletion.** Female DEREG mice received 1 µg DT solute i.v. on two consecutive days before readout or CLP. Control mice received PBS. Splenocytes (A) and blood leukocytes (B) were analysed by flow cytometry for Foxp3 expression in CD4^+^ T cells. Cryosections from mesenteric lymph node (C) and thymus (D) were analysed for CD4 (red) and Foxp3 (green) expression with a fluorescence microscope. The DT treatment ablated around 95% of the Foxp3^+^ T cells. Results from one representative animal are shown.
(PPTX)

Figure S2 **Purity of the isolated Tregs, Teffs and APCs.** The increased purity of Tregs and Teffs after MACS column-based negative CD4^+^ T cell enrichment and flow cytometric sorting is shown in A. Foxp3^+^ Treg preparations were about 98% pure, 70% of the cells were viable (B). Foxp3^−^ Teffs had a purity of 95% with a viability of 94% (B). APCs were sorted with MACS columns based on CD11c. Preparations were 90% CD11c^+^ cells (C). For purity and viability staining, the following reagents were used: CD4-Alexafluor647 (Gk1.5), CD11c-FITC (HL3) and an AnnexinV-PE apoptosis detection kit from BD Biosciences.
(PPTX)

Figure S3 **CD28 SA-mediated activation of Tregs in vivo.** DEREG mice received 250 µg CD28 SA i.v. three days before their spleens were explanted and CD4^+^Foxp3^+^ Tregs isolated. Control mice were left untreated or received an isotype control antibody (MOPC-31C). CD28 SA treatment increased the numbers of splenic Tregs and Teffs (A) and increased CTLA-4 expression by Tregs (B). To test for functional activity (C), 5×10^4^ untreated CD4^+^Foxp3^−^ Teffs were co-incubated at the given ratio with CD4^+^Foxp3^+^ Tregs that were either untreated (open bars) or pre-activated with CD28SA *in vivo* (filled bars). They were co-cultured for 72 h with 1×10^4^ irradiated APC and 1 µg/ml soluble anti-CD3 antibody. ^3^H-Thymidine was added for the last 17 hours of culture. ^3^H-Thymidine incorporation (cpm) of Teffs without Tregs was set to 100%. *In vivo* CD28 SA treatment markedly increased the suppressive potential of the Tregs. One out of two experiments with similar results is depicted. Means +/− SEM are shown.
(PPTX)
